# Unprecedented Finding of Isolated Sphenoid Fungal Ball in a Child: A Case Report

**DOI:** 10.1155/crot/4404155

**Published:** 2025-07-14

**Authors:** Karl El Mendelek, Joseph Makhlouf, Charbel Daoud, Fouad El Fata, Jihad El Khoury

**Affiliations:** Department of Otolaryngology—Head and Neck Surgery, Saint George Hospital University Medical Center, Beirut, Lebanon

**Keywords:** fungal ball, headache, mycetoma, sphenoid sinus

## Abstract

Sphenoid sinus fungal ball is an uncommon disease that often occurs in adult patients, due to the rarity of sphenoid diseases in the pediatric population. To our knowledge, we were able to describe the first case of isolated sphenoid fungal ball, or mycetoma, in a child. No previous cases have been mentioned in the literature. We are presenting the case of a pediatric patient who presented with a long-standing refractory headache.

## 1. Introduction

Isolated sphenoid sinus disease (ISSD) is a broad term referring to a large number of diseases where acute or chronic inflammation of the sphenoid sinus mucosa is typically present. It is considered an uncommon entity, accounting for less than 3% of all paranasal sinus infections [1], probably due to the anatomical and topographic characteristics of the sphenoid sinus where pathology usually progresses from other sinuses [2, 3]. On the other hand, the physiology of the sphenoid mucosa containing relatively few mucous-secreting cells makes it a rarer ground for opacification and filling when compared to the other paranasal sinuses. Whether ISSD is caused by bacterial, fungal, tumorous, allergic, or inflammatory conditions, the clinical presentation is commonly misleading due to the predominance of headache and visual disturbances, sometimes lacking the typical nasal symptoms of acute or chronic rhinosinusitis [4]. Therefore, the diagnosis is usually challenging and delayed. Sphenoid sinusitis is particularly rare in children because the pneumatization of the sphenoid sinus starts at 2-3 years of age, making sphenoid infections usually apparent only after the age of 5 years [5, 6].

Fungal sinusitis is commonly classified as being invasive or noninvasive, depending on the presence or absence of soft tissue invasion and angioinvasion on histopathology. Fungal ball, also known as mycetoma or aspergilloma, is a noninvasive fungal infection most frequently caused by Aspergillus species and confined to the paranasal sinuses [7]. However, bone erosion is not an uncommon finding in advanced disease. Fungal hyphae usually accumulate in a single sinus, affecting the maxillary sinus most commonly, followed by the sphenoid, ethmoidal, and frontal sinuses in that order. Mycetoma is confined to the sphenoid sinus in 4.5%–26.8% of fungal ball cases [4].

## 2. Case Report

A 10-year-old male patient, with no significant past medical history or known food or drug allergies, presented to the otolaryngology clinics with headache. History went back to 2 years prior to presentation when he started experiencing occasional episodes of generalized headache which were not related to any obvious etiological factor, according to his parents. No other symptoms were reported at that time. The episodes increased in frequency and intensity over time, affecting some daily life activities and school performance. A couple of months later, he started describing the pain as retro-orbital, especially during attention activities.

**Figure 1:**
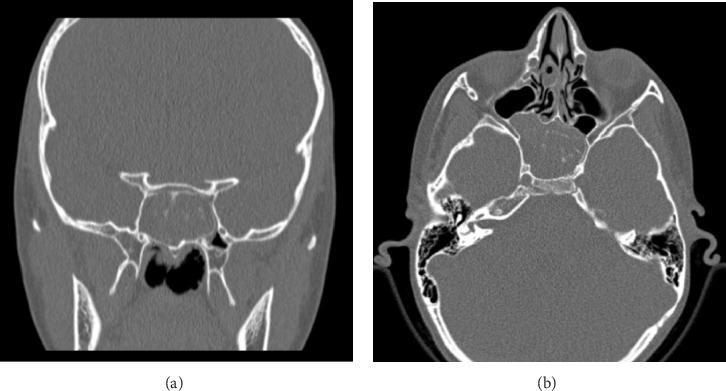
Brain CT showing complete opacification of the sphenoid sinus with erosion of the sphenoid septum and multiple hyperdense deposits: coronal view (a); axial view (b).

The patient was initially taken to his pediatric physician who attributed his symptoms to “ocular strain” and subsequent muscular tension and referred him to an optician. Eyeglasses for mild degree of astigmatism were designed for the patient at that time. However, his symptoms persisted, so he was seen and examined by a pediatric neurologist 1 year later, who diagnosed him with migraine. He was prescribed ibuprofen to be taken as needed for symptomatic control. Nevertheless, no significant improvement was noted. A computed tomography (CT) scan of the brain was then ordered in order to rule out any intracranial etiology of the refractory headache. Surprisingly, complete opacification of the sphenoid sinus with foci of calcific deposits was noted (Figure 1).

**Figure 2:**
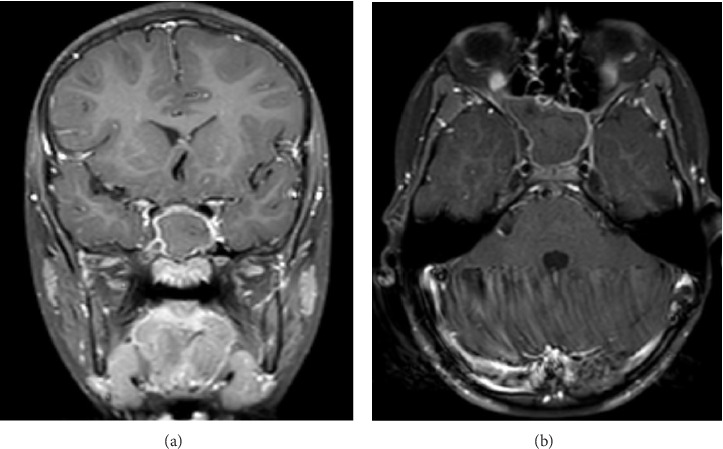
Brain MRI showing sphenoid lesion on T1 sequence with IV gadolinium: coronal view (a); axial view (b).

In front of these findings which raised the concern of an unprecedented occurrence of sphenoid sinus fungal ball in a pediatric patient, further workup was requested. Upon additional history taking, parents denied any current illness or history of immunosuppressive therapy. They also denied any prior dental procedures, except for regular and uncomplicated baby teeth extraction. The patient was not taking any medications. A magnetic resonance imaging (MRI) of the brain with intravenous (IV) gadolinium was done for better characterization of the sphenoid lesion (Figure 2).

Similarly, MRI results showed complete filling of the sphenoid sinus with a hypo- to isointense content, and enhancement of the sinus mucosa with IV contrast which is in favor of chronic inflammation associated with long-standing fungal ball.

The patient was finally referred to the otolaryngology department. Examination of the nasal cavity with anterior rhinoscopy followed by flexible nasal endoscopy did not show any evidence of purulent drainage, mucosal irritation, or congestion. On physical examination, cranial nerves were intact. Inspection of the facial features and oropharyngeal cavity, as well as otoscopic evaluation of the ears, was all normal. The treatment plan was discussed with the family, and the patient underwent a sphenoid sinusotomy without any perioperative complication. A thick, foul-smelling, and clay-like material was removed from the sphenoid sinus and sent for histopathological analysis. Postoperative prophylactic dose of amoxicillin-clavulanate adjusted to the patient's weight was given for 1 week, as well as nasal saline irrigation for 1 month, to promote proper mucosal healing. The specimen sent for histopathological examination showed evidence of chronic mucosal disease and fungal hyphae confined to the sinus debris not invading adjacent blood vessels, bone, or soft tissue, in favor of fungal ball. Fungal culture showed no growth after 1 week of incubation. No bacterial or mycobacterial pathogens were isolated.

**Figure 3:**
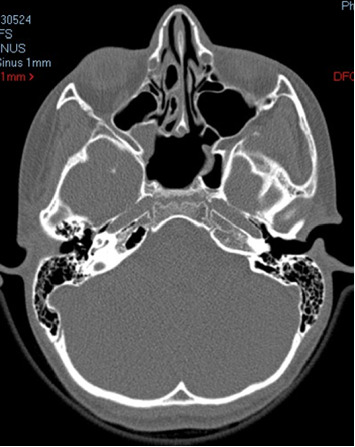
Brain CT in axial view with complete aeration of the sphenoid sinus.

The patient was seen and re-examined 3 months after the surgery; the results were excellent. There was complete remission from the initial symptoms. Repeated CT scan of the brain is shown in Figure 3. It shows complete aeration of the sphenoid sinus with resolution of the disease.

## 3. Discussion

The sphenoid sinus is a particularly delicate topography for diseases due to its proximity to many vital structures, including the cavernous sinus where cranial Nerves III, IV, V1, V2, and VI lie, as well as the pituitary gland, the internal carotid artery, and the optic chiasm [4]. Our patient had a noninvasive type of fungal sphenoid sinusitis where infection and inflammation were confined to the bony walls. He had normal extra-ocular muscle movements and normal sensation over the entire face bilaterally and did not show any signs or symptoms of hypopituitarism or focal neurological deficit. It is important to note that there have been few reports that have shown that sinus fungal balls could progress into chronic invasive fungal sinusitis in less than 2% of cases, especially in immunocompromised patients [8, 9]. The absence of extension into nearby structures in our patient was confirmed by brain imaging, even though it was performed at a relatively late stage after disease onset.

In their updated review, Akhondi et al. have found that aspergillomas almost always involve the maxillary sinus [10]. It usually starts after inhalation of fungal spores. Epidemiological analyses showed that in most cases, it affects female patients with a mean age of onset above 40 years and is unilateral in presentation [10–12]. This form of fungal infection occurs in immunocompetent hosts who lack atopic features, as opposed to allergic fungal rhinosinusitis (AFRS) which is the most common type of noninvasive fungal sinusitis with usually bilateral involvement [12]. AFRS was ruled out in our case because the patient did not have any history of atopy such as asthma or other allergies, his disease was well confined as described on CT scan, and there was no evidence of sinonasal polyps or secretions (eosinophilic mucin) on nasal endoscopy. In other words, the Bent and Kuhn diagnostic criteria of AFRS were not met.

Uncomplicated sphenoid sinus fungal balls most commonly present initially with purulent anterior or posterior rhinorrhea, nasal obstruction, and hyposmia [4]. These nasal symptoms can be primary or secondary to a superimposed bacterial infection caused by obstruction of the sphenoethmoidal recess by the fungal hyphae [9]. Interestingly, our patient did not experience any nasal symptom throughout 2 years of infection. Headache is another well-known important hallmark of the disease [13] and was the only complaint reported by our patient. In some cases of maxillary sinus mycetoma, the disease is asymptomatic and discovered incidentally on imaging performed for another reason [14]; this is usually not the case with sphenoid aspergillomas. All symptomatic paranasal fungal balls require complete evacuation with functional endoscopic sinus surgery (FESS), which is the gold standard therapeutic approach [13, 15]. Treatment of asymptomatic and incidentally discovered lesions remains debatable, ranging from simple observation to surgical removal due to the minimal risk of progression into invasive disease in case of acquired immunosuppression.

In contrast to the abovementioned characteristics of fungal balls, the case described in our paper was unique in that the disease solely involved the sphenoid sinus, with completely sparing of the maxillary sinuses. Scattered reports have described the incidence of isolated sphenoid fungal balls in the adult population [1, 2, 4, 5], but to our knowledge, we were able to describe the first case of isolated sphenoid mycetoma in a pediatric patient, noting that isolated sphenoiditis in children, regardless the etiology, is extremely rare [6]. Kotowski and Szydlowski described in their cohort study some cases of ISSD in the pediatric population, including mucoceles, lipomas, osteomas, and polyps; none of which corresponded to a fungal etiology [6].

## 4. Conclusion

Fungal balls confined to the sphenoid sinus are an interesting field of research due to their uncommonness. After being described in a pediatric patient in our report, sphenoid mycetoma should become an additional differential diagnosis in a child presenting with unresolving nasal symptoms, or more exceptionally, refractory headache. Risk factors for isolated sphenoid fungal infections are not clearly described in the literature, and hence cannot contribute to the physician's index of suspicion when diagnosing or treating a child with long-standing headache. Endoscopic sinus surgery with complete evacuation is the mainstay of treatment, and follow-up is strongly recommended to rule out disease recurrence which is strongly unusual.

## Data Availability

The data that support the findings of this study are available from the corresponding author upon reasonable request.
